# Association Between Hyponatremia and Mortality and Readmission in Multimorbid Older Adults—A Cohort Study

**DOI:** 10.3390/jcm14207146

**Published:** 2025-10-10

**Authors:** Seraina Netzer, Viktoria Gastens, Benoît Boland, Carole E. Aubert, Corlina J. A. Huibers, Wilma Knol, Anne Spinewine, Denis O’Mahony, Drahomir A. Aujesky, Mirjam Christ-Crain, Douglas C. Bauer, Nicolas Rodondi, Martin Feller

**Affiliations:** 1 Department of General Internal Medicine, Inselspital, Bern University Hospital, University of Bern, 3010 Bern, Switzerland; 2 Institute of Primary Health Care (BIHAM), University of Bern, 3010 Bern, Switzerlandmartin.feller@extern.insel.ch (M.F.); 3 Laboratory of Population Health, University of Fribourg, 1700 Fribourg, Switzerland; 4 Department of Geriatric Medicine, UC Louvain, St-Luc University Hospital, 1200 Brussels, Belgium; 5 Institute of Health and Society (IRSS), Université Catholique de Louvain, 1200 Brussels, Belgium; 6 Department of Geriatric Medicine, University Medical Centre Utrecht, Utrecht University, 3508 TC Utrecht, The Netherlands; 7 Department of Pharmacy, CHU UCL Namur, 5530 Yvoir, Belgium; 8 Louvain Drug Research Institute, Université Catholique de Louvain, 1200 Brussels, Belgium; 9 Department of Medicine, School of Medicine, University College Cork, T12AK54 Cork, Ireland; 10 Department of Geriatric Medicine, Cork University Hospital, Wilton, T12DC4A Cork, Ireland; 11 Department of Clinical Research and Department of Endocrinology, Diabetes and Metabolism, University Hospital Basel, University of Basel, 4031 Basel, Switzerland; 12 Department of Medicine and Department of Epidemiology and Biostatistics, University of California, San Francisco, CA 94158, USA

**Keywords:** hyponatremia, multimorbidity, older adults, mortality, hospital readmission

## Abstract

**Background/Objectives**: Hyponatremia has been associated with mortality and hospital readmissions. Although multimorbid older patients are particularly affected, specific data on this group are lacking. **Methods**: A prospective cohort was used based on the OPERAM (OPtimising thERapy to prevent Avoidable hospital admissions in the Multimorbid elderly) trial, a European multicenter, cluster-randomized trial among hospitalized patients aged ≥70 years with ≥3 chronic medical conditions taking ≥5 long-term medications, with documented sodium values at admission, excluding participants with hypernatremia (>145 mmol/L). The primary outcome was all-cause 1-year mortality, and secondary outcomes were 30-day mortality and readmission at 1 year and at 30 days. We examined the association between hyponatremia and mortality in comparison to normonatremia using a mixed-effects survival model, with adjustment for age, sex, comorbidities, study intervention arm, study site and cluster; and the association between hyponatremia and readmission using competing risk models with death as the competing risk. Subgroup analyses were performed across sodium hyponatremia categories (mild 134–130 mmol/L, moderate 129–125 mmol/L, severe < 125 mmol/L). **Results**: Of 2008 OPERAM participants, 1968 had a sodium value at admission, and 33 were excluded due to hypernatremia. In the 1935 participants, the mean age was 79.4 years (standard deviation 6.3), 866 (44.8%) were female, the median number of comorbidities was 11 (IQR 8–16), the median number of drugs was 10 (IQR 7–13), and 401 (20.7%) had hyponatremia at admission. The multivariate-adjusted hazard ratio (HR) for 1-year mortality with hyponatremia was 1.41 (95% confidence interval [CI] 1.11–1.78, 364 deaths) and for 30-day mortality was 1.20 (95%CI 0.74–1.94, 89 deaths). The adjusted sub-HR for 1-year readmission was 0.94 (95%CI 0.79–1.11), and that for 30-day readmission was 1.1 (95%CI 0.78–1.59). There was a linear increase in 1-year mortality across hyponatremia categories (HR from 1.31 to 2.64, *p* for trend = 0.001). **Conclusions**: Hyponatremia at admission is associated with increased 1-year mortality in multimorbid older adults, with increasing risk for lower sodium values. These findings support sodium as a useful prognostic marker in this setting, while its potential independent impact on survival remains to be clarified in prospective studies.

## 1. Introduction

Hyponatremia, defined by a sodium value < 135 mmol/L, is the most common electrolyte disorder. Prevalence estimates vary by setting: while hyponatremia affects < 10% of community-dwelling adults, it is found in up to one third of hospitalized patients and is even more frequent in nursing home residents and those receiving long-term care [1,2,3]. It is commonly even further categorized into mild (130–134 mmol/L), moderate (125–129 mmol/L) and severe (<125 mmol/L) hyponatremia [4]. Underlying causes of hyponatremia are manifold and include several age-related illnesses, such as heart failure, chronic kidney disease, pulmonary disorders and malignancies [5,6,7,8]. Numerous drugs, such as thiazides and antidepressants, can also cause hyponatremia, with mounting risk with concomitant use and polypharmacy [9,10,11].

The underlying mechanism of hyponatremia is most commonly by way of increased antidiuretic hormone (ADH) levels, which in turn leads to impaired ability to excrete ingested water [12]. Older adults can experience higher levels of ADH due to age-related changes in homeostatic mechanisms, which often also include decreased urinary concentrating ability, glomerular filtration rate and aldosterone levels, further increasing the likelihood of hyponatremia [13]. Consequently, multimorbid older adults with polypharmacy are at particular risk.

Clinical manifestations of hyponatremia depend on severity and whether it is acute (<48 h) or chronic (>48 h). While mild chronic hyponatremia can be asymptomatic, acute severe hyponatremia can lead to seizures, coma and death [1,10]. Further possible manifestations range from subtle cognitive changes to gait disturbances, falls and fractures [14,15,16,17]. In older adults, such complications are of special concern as falls and fractures can lead to loss of independence, institutionalization and higher long-term mortality [14]. Even mild chronic hyponatremia has been linked with impaired attention, increased risk of delirium, prolonged hospital stays and elevated healthcare costs [18].

Additionally, several studies have shown an association between low sodium and in-hospital mortality [19,20]. However, there is a distinct lack of studies focusing on multimorbid older adults, and studies with follow-up beyond hospitalization are rare, with conflicting results [21,22,23]. To address this knowledge gap, we investigated the association of hyponatremia with mortality and hospital readmission in a large European cohort of hospitalized multimorbid older adults.

## 2. Materials and Methods

### 2.1. Study Design, Setting and Population

The present study was a prospective cohort analysis based on the OPERAM (OPtimising thERapy to prevent Avoidable hospital admissions in Multimorbid older adults) data [24]. OPERAM was a European multicenter, cluster-randomized controlled trial conducted in four university hospitals (Bern, Switzerland; Utrecht, The Netherlands; Louvain, Belgium; and Cork, Ireland). Details of the trial design have been published elsewhere [25,26]. In brief, clusters were defined at the level of attending hospital doctors, who were randomized 1:1 to either a structured pharmacotherapy optimization intervention or usual care. The intervention consisted of a physician–pharmacist team performing a systematic medication review using the STRIP/STRIPA tool (ExpertDoc B.V., Amsterdam, The Netherlands), while the control arm received usual care and a sham intervention to preserve blinding. Eligible participants were adults aged ≥70 years admitted to hospital with multimorbidity (≥3 chronic conditions) and polypharmacy (≥5 long-term medications). Exclusion criteria were minimal to ensure generalizability, limited to planned transfer to palliative care within 24 h, recent participation in another structured drug review or inability to provide consent (or obtain proxy consent). Between December 2016 and October 2018, 2008 participants were enrolled and followed for 12 months. Follow-up data were collected through telephone interviews with the participants or their proxies at 2, 6 and 12 months. The main OPERAM trial showed no effect of the intervention on drug-related readmissions, falls and mortality at 1 year.

For this study, the inclusion criterion was the availability of sodium values at admission for the index hospitalization. Participants with sodium values consistent with hypernatremia (sodium > 145 mmol/L) were excluded from the analysis. Sodium values 135–145 mmol/L were considered normal; <135 mmol/L was considered as hyponatremia [4]. For subgroup analyses, we further stratified hyponatremia into mild (130–134 mmol/L), moderate (125–129 mmol/L) and severe (<125 mmol/L) [1,20]. We defined sodium trajectories for participants who had a second sodium measurement available at discharge as follows: normonatremia (two normal values 135–145 mmol/L), persistent hyponatremia (two sodium values < 135 mmol/L), resolved hyponatremia (hyponatremia at admission, normal sodium at discharge) and acquired hyponatremia (normal sodium at admission, hyponatremia at discharge).

### 2.2. Outcomes

For the main analysis, death at one year was the primary outcome; secondary outcomes were death at 30 days and hospital readmission at one year and at 30 days. Exploratory outcomes were hospital readmissions due to falls and fractures.

### 2.3. Statistical Analysis

We described baseline characteristics separately for the hyponatremic and normonatremic participants. For the outcomes of death at one year and 30 days, we used a mixed survival model with adjustment for age, sex, comorbidities using the weighted Charlson comorbidity index [27], the study site and the study arm, with the cluster, based on the treating physician during the index hospitalization, as a random effect. We displayed the results for the main outcome graphically with a Kaplan–Meier survival function. For hospital readmission at one year and 30 days, we used a competing risk model with the same adjustment variables as mentioned above, with death as a competing risk. The proportional hazards assumption was assessed graphically using log–log survival curves adjusted for age, sex and comorbidity score. In addition, we stratified the main results according to the severity of hyponatremia (mild, moderate, severe) and tested for linear trends across the sodium categories.

We performed five sensitivity analyses: (i) We adjusted for common causes of hyponatremia that could significantly influence mortality, namely the presence of heart failure [28], any malignancy [8,29], metastasized solid tumors [30] and infection [31] during index hospitalization (for utilized ICD-10 codes for infection, see Supplementary Materials Table S1) [32]. (ii) We restricted the study population to those not taking drugs known to have potentially relevant effects on sodium values, as drug-related hyponatremia might differ in prognosis to other causes and to assess whether the association between hyponatremia and outcomes was independent of such medications (see Supplementary Table S2 for a list of excluded drugs) [33]. (iii) In a third sensitivity analysis, we acknowledged that hyperglycemia can cause pseudohyponatremia, which could bias our results [34]. Unfortunately, glucose was not systematically recorded. To explore the potential extent of this misclassification, we therefore randomly selected (via computer generation) 100 participants from Switzerland and retrieved serum glucose levels. We then repeated the main analyses with glucose-corrected sodium values (using the Hillier formula) [34]. (iv) We restricted the analysis to participants with two sodium measurements during the index hospitalization specifically at baseline and at discharge from the index hospitalization, in order to assess in-hospital changes in sodium. Only these two time points were systematically recorded, irrespective of how many sodium measurements were performed clinically during the hospitalization. (v) We further adjusted the analysis from (iv) for the sodium changes, accounting for the hypothesis that participants with persistent or acquired hyponatremia during index hospitalization may suffer worse outcomes.

In exploratory analyses, we examined whether all-cause mortality and hospital readmission (both at 1 year and at 30 days) differed across sodium trajectories, using the same statistical models as for the main analyses, i.e., mixed survival models for death, and competing risk models for readmissions. We performed pairwise comparisons of marginal linear predictions using Bonferroni correction. Additionally, we repeated the primary analyses with the sodium measurement at discharge, as most acute causes of hyponatremia might well be resolved at discharge. In another exploratory analysis predefined in the published protocol, we assessed whether hyponatremia is associated with an increased risk of readmission due to falls or fractures. This outcome was chosen based on prior reports linking hyponatremia to falls and fractures [14,15,35], and we considered it a potential explanation for hypothetically higher readmission rates. For fractures, we performed additional sensitivity analysis adjusting for prescription of bisphosphonates (proxy for osteoporosis). The rationale was to examine if hyponatremia was associated with fractures independently of osteoporosis.

In post hoc sensitivity analyses we modeled sodium as a continuous variable, in a second step using restricted cubic splines for the primary outcome, with five knots placed at Harrell-style percentiles of 5, 27.5, 50, 72.5 and 95 (129, 136, 138, 140 and 143 mmol/L).

Statistical analyses were performed using STATA/MP version 16.1 (StataCorp LLC, College Station, TX, USA) for Windows.

## 3. Results

Of 2008 OPERAM participants, 1968 (98.1%) had sodium measurement at admission. A total of 33 participants (1.6%) had an elevated sodium value (>145 mmol/L) and were excluded, thus leaving 1935 participants (96.4%) for inclusion in the analyses (Table 1).

### 3.1. Mortality and Readmission Overall and Across Sodium Categories

**Figure 1:**
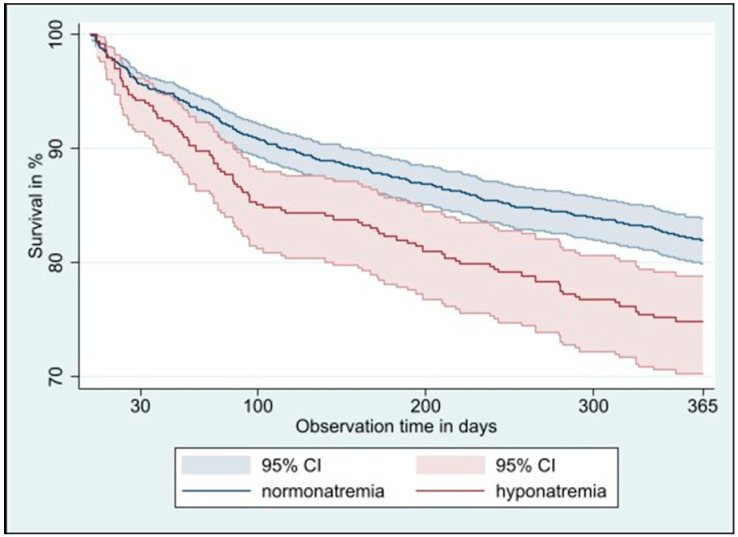
Kaplan–Meier 1-year survival curve in multimorbid older adults with normonatremia versus hyponatremia at hospital admission. Abbreviations: CI: confidence interval.

After one year, 364 participants (18.8%) of the 1935 included participants had died, 98 (24.4%) of whom were among the 401 participants with hyponatremia and 266 (17.3%) of whom were among the 1534 participants with normal sodium values at admission. Checks of the proportional hazards assumption did not indicate relevant deviations, suggesting that the assumption was reasonably met. After adjustment for age, sex, comorbidities using the weighted Charlson comorbidity index, intervention, study site and cluster as a random effect, risk of mortality at one year was significantly higher for participants with hyponatremia with a hazard ratio (HR) of 1.41 (95% confidence interval (CI) 1.11–1.78, *p* = 0.005, Figure 1), with a linear increase across sodium categories from mild to moderate to severe (*p* for trend = 0.001). After 30 days, we found no statistically significant higher mortality in participants with hyponatremia at admission (HR 1.20, 95% CI 0.74–1.94, *p* = 0.46), but the risk increased significantly across sodium categories (*p* for trend = 0.012). Hospital readmission did not differ between participants with hyponatremia and those with normal sodium values at one year (subhazard ratio (SHR) 0.94, 95% CI 0.79–1.11, *p* = 0.46) or at 30 days (SHR 1.11, 95% CI 0.78–1.59, *p* = 0.55), with no difference in risk across sodium categories (Table 2).

### 3.2. Sensitivity Analyses

#### 3.2.1. Common Causes of Hyponatremia

Repeating the main analysis with further adjustment for the presence of heart failure, any malignancy, metastasized solid tumors or infection during the index hospitalization did not significantly change the results for mortality or readmission at either 30 days or one year (see Supplementary Table S3).

#### 3.2.2. Drugs

In a sensitivity analysis, 866 participants who took potentially hyponatremia-inducing drugs were excluded. In the remaining 1069 participants, 212 (19.8%) had hyponatremia, 215 (20.1%) died within one year and 508 (47.5%) were readmitted to the hospital within one year. Risk of all-cause mortality at one year was higher for participants with hyponatremia at admission, with an HR of 1.51 (95% CI 1.10–2.05, *p* = 0.01). At 30 days, we found a trend towards increased risk of mortality for participants with hyponatremia compared to participants with normal sodium (HR 1.57, 95% CI 0.89–2.79, *p* = 0.12). Hospital readmission risk did not differ between participants with hyponatremia and those with normal sodium values at one year (SHR 0.92, 95% CI 0.72–1.17, *p* = 0.49) or at 30 days (SHR 1.00, 95% CI 0.64–1.56, *p* = 0.99).

#### 3.2.3. Glucose

In a random sample of 100 Swiss participants with blood glucose values, mean glucose was 7.51 mmol/L (135 mg/dL), the standard deviation (SD) was 2.77 mmol/L (50 mg/dL), the range was 4.0–17.8 mmol/L (72–321 mg/dL) and mean sodium was 138.4 mmol/L, with 15 participants with hyponatremia. After glucose correction, mean sodium was 139.2 mmol/L, and three participants (3%) were reclassified from hyponatremia to normal sodium values. Of these three, two participants had only slightly elevated glucose values (7.6 mol/L (137 mg/dL) and 9.8 mmol/L (177 mg/dL), respectively) and a sodium value of 134 mmol/L before and 135 mmol/L after adjustment. Repeating the main analysis with the adjusted sodium values did not significantly change the results.

#### 3.2.4. Sodium Changes

A total of 1903 participants (98.3%) had sodium values measured at admission and at a second time point. Of these, 60 (3.2%) had hypernatremia (>145 mmol/L) at the second measurement and were therefore excluded from the analysis of sodium trajectories. For a further 61 participants (3.2%), the second sodium measurement documented was measured during another hospitalization and they were therefore also excluded from the analysis. Overall, 1782 participants (92.1%) had sodium values consistent with either normal sodium or hyponatremia at admission and discharge. The mean time between sodium measurements was 7 days (median 5, IQR 2–9). In these participants with two sodium measurements, hyponatremia at admission was associated with a significantly higher mortality risk at one year than normal sodium (HR 1.45, 95% CI 1.12–1.87, *p* = 0.004). Repeating the main analysis with additional adjustment for the change in sodium yielded similar results for 1-year mortality (HR 1.49, 95% CI 1.11–2.01, *p* = 0.008) and 30-day mortality (HR 1.37, 95% CI 0.59–3.18, *p* = 0.46). Adjusting for change in sodium did not increase the risk of 1-year readmission (SHR 0.74, 95% CI 0.58–0.95, *p* = 0.02) or 30-day readmission (SHR 1.10, 95% CI 0.72–1.66, *p* = 0.68).

### 3.3. Exploratory Aims

#### 3.3.1. Sodium Trajectories

Observing trajectories, mortality risk at one year was higher for participants with persistent hyponatremia, but not for those with resolved and acquired hyponatremia, compared to participants with two normal sodium values (Table 3). However, pairwise comparisons using Bonferroni correction did not show significant differences between the hyponatremia trajectory groups, thus showing no evidence that the subgroups were significantly different from each other. Observing trajectories, mortality risk at 30 days did not differ from normal serum sodium, without differences in the pairwise comparisons. Hyponatremia on at least one measurement did not raise the risk of readmission at one year (SHR 0.81, 95% CI 0.67–0.99, *p* = 0.04) or at 30 days (SHR 1.25, 95% CI 0.87–1.79, *p* = 0.23). This did not differ when testing for sodium trajectories.

#### 3.3.2. Sodium at Discharge

Repeating the main analysis with the sodium measured at discharge did not significantly change the results for mortality or readmission at either 30 days or one year (see Supplementary Table S4).

#### 3.3.3. Falls and Fractures

Overall, 264 participants (13.6% of all, 27.6% of participants with readmissions) had hospital readmissions due to falls. Participants with hyponatremia were not more likely to be readmitted to a hospital due to falls (15.5% of participants with hyponatremia, 13.2% of participants with normal sodium, *p* = 0.23/SHR 1.00, 95%-CI 0.72–1.38, *p* = 0.99). In 113 participants (5.8%), falls led to fractures. This was not more likely in participants with hyponatremia (6.5% vs. 5.7%, *p* = 0.54/SHR 0.98, 95%-CI 0.63–1.52, *p* = 0.92). The results did not change when adjusted for bisphosphates as a proxy indicator of osteoporosis (SHR 0.98, 95%-CI 0.63–1.53, *p* = 0.93).

### 3.4. Post Hoc Analyses

In continuous analyses, each 1 mmol/L higher sodium was associated with a 4% lower hazard of death (HR 0.96, 95% CI 0.94–0.98). Restricted cubic spline analysis demonstrated a monotonic association between lower sodium and higher mortality risk: mortality increased progressively below 135 mmol/L, while the curve flattened within the normal sodium range (135–145 mmol/L) (see Supplementary Figure S1). Change in sodium did not change the risk for readmission (SHR 1.00, 95% CI 0.99–1.02).

## 4. Discussion

In this cohort study of multimorbid older adults, participants with hyponatremia at hospital admission had 41% higher mortality within one year compared to participants with normal sodium at admission, but not at 30 days. Further, hyponatremia at admission was not associated with 1-year or 30-day readmission.

There are several unanswered questions on the prognostic impact of hyponatremia. Stress from an illness activates the hypothalamic–pituitary–adrenal axis, which in turn increases vasopressin secretion, which leads to loss of sodium [36]. Hyponatremia could thus simply be a sign of an organism under duress. Here, the underlying cause is the prognostically significant factor. But it is also possible that hyponatremia itself has a negative impact on survival due to the adaptive measures the human body takes when suffering from hyponatremia, called allostasis [36]. The exact processes are still poorly understood. One potential mechanism was identified in animal models, which showed a concomitant decrease in taurine in hyponatremia [37]. Taurine is one of the most prevalent osmolytes in the cardiomyocites and protects them against apoptosis [38]. Furthermore, disturbances in the osmotic equilibrium as with hyponatremia can lead to cell swelling or shrinkage and thus cellular dysfunction, with neurons being particularly vulnerable to osmotic shifts. Taken together, these findings suggest that hyponatremia may act both as a marker of underlying illness and as a potential direct contributor to adverse outcomes.

Several large studies have found an association between low sodium and in-hospital mortality [19,20,39]. The largest study, with over 2 million participants, of which roughly 360,000 had hyponatremia at admission, showed an association between hyponatremia and in-hospital mortality, which diminished with increasing age [19]. While there are several large retrospective studies on hyponatremia and its association with mortality beyond hospital admission [39,40,41,42], none has focused specifically on multimorbid older adults. Four studies focused on older participants and reported conflicting results. In a Chinese study of 4364 participants aged 80 years or older hospitalized in one tertiary-level hospital, the authors observed an association between hyponatremia <130 mmol/L and hospital length of stay as well as in-hospital mortality [20]. However, this was a single-center study, did not follow up after hospital discharge and compared hyponatremia categories but did not compare to normal sodium values. In a prospective Greek study of 138 participants aged 65 years or older with hyponatremia <130 mmol/L at admission to a tertiary hospital, the authors observed an increased in-hospital mortality compared to patients admitted in the same time frame with normal sodium values (17.4% vs. 13.4%) [22]. They followed mortality and readmission rates of the hyponatremic patients for one year post-index admission, but they lacked a comparative normonatremic control group for the follow-up after hospitalization. In a Dutch study of 945 patients aged 65 years or older admitted to one tertiary hospital, 307 (34.3%) patients had hyponatremia on admission. The authors examined the association between hyponatremia and both in-hospital and 3-month mortality and found no association after adjusting for comorbidities [21]. However, the study was single-center and the methodology was barely reported, with missing information, thus leaving the possibility of bias. In a German study of 141 patients with sodium values <130 mmol/L admitted to one tertiary hospital and 141 controls (matched for age, sex and admission diagnosis), the authors did not find an increased risk of mortality at 6 months in the hyponatremic group compared with the normonatremic group [23]. However, this was once again a single-center study and the results were not adjusted for relevant comorbidities and medication.

A further noteworthy study is the recent large-scale propensity-matched cohort analysis from Stockholm by Mannheimer et al. [42]. Using a pharmacoepidemiologic database covering 1.6 million individuals, they compared nearly 67,000 hospitalized patients with hyponatremia at admission to matched normonatremic controls. They found that 30-day mortality increased stepwise with greater severity of hyponatremia (HRs of 1.35 for mild to 3.38 for very profound), while one-year mortality remained elevated in moderate and more severe cases. In patients with mild hyponatremia, excess mortality largely disappeared after the first month, consistent with a “harvesting” phenomenon, where short-term deaths reflect frailty and acute illness rather than sodium itself. Only sodium at admission was taken into account. While this cohort was not restricted to multimorbid older adults with polypharmacy, this population was also represented, although not analyzed separately. Their findings support our results by suggesting that hyponatremia is both a marker of underlying disease severity and a potential independent contributor to mortality, the latter especially at lower sodium levels.

In the present study, we found a significantly increased adjusted risk of mortality at one year after index hospital admission for participants with hyponatremia, but not at 30 days. The outcome of 30-day mortality (OR 1.16, 95%CI 0.72–1.87, *p* = 0.47, number of deaths at 30 days = 89) was underpowered. Thus, this change in risk is most likely a reflection of the smaller number of events that did not allow an earlier detection of a significant association. This association could be due to the underlying cause of hyponatremia, but our results did not differ when adjusting for comorbidities or medication. While it is possible that we missed a relevant unknown confounder, an independent association between hyponatremia and mortality could also be due to allostasis, i.e., adaptive human physiological mechanisms as a response to hyponatremia, as discussed above [36].

In subgroup analyses, mortality increased across hyponatremia categories from mild to severe, which is in accordance with previously published data [39,41,42]. In a further subgroup analysis for sodium trajectories, we found an increased association for 1-year mortality in participants with persistent hyponatremia and some evidence of an increased association with acquired and resolved hyponatremia. Comparing the groups pairwise did not show significant differences, hence we have no clear evidence that the subgroups were significantly different from each other. For 30-day mortality, no trajectory group showed a higher risk, but participant numbers were very small (e.g., 57 with acquired hyponatremia, two deaths within 30 days in that group). Furthermore, pairwise comparison did not show significant differences between the groups. Previous observational studies including participants with mild hyponatremia also showed an association with mortality, regardless of sodium trajectory when compared to normonatremia [41,43]. In contrast, a previous meta-analysis found an association between hyponatremia improvement and reduced mortality risk, but was mainly based on studies that included only participants with moderate to severe hyponatremia and were not focused on multimorbid older adults [44].

We found no evidence for higher risk of readmission for participants with hyponatremia regardless of time point (30 days or 1 year), sodium categories, trajectories or time of sodium measurement (admission or discharge), with even a slightly lower risk for admission at one year in those with hyponatremia at discharge. It is surprising that our data show a strong association between hyponatremia and increased mortality, but not readmission. A possible explanation is that either the deaths occurred too quickly to allow for hospitalization, or the deaths occurred in other settings such as nursing homes, hospices or homes, without a transfer back to hospital, which could be the case, for example, for end-stage cancer patients.

A possible mechanism for hyponatremia to cause readmission is increased falls due to symptomatic hyponatremia. As we had assumed to find an association between hyponatremia and readmission, we had predefined falls and fractures as an exploratory aim. However, falls and fractures as reasons for readmission were not more common in participants with hyponatremia than in participants with normonatremia. This finding contrasts with previous studies that show an association between hyponatremia and falls with fractures [15,16]. However, the sodium concentrations at the time of readmission in our participants were not routinely documented and thus we do not know if these participants were hyponatremic at the time of their falls or fractures.

Our study has several limitations. First, we did not have serum osmolality or volume status data on trial participants and therefore could not determine whether hyponatremia was hypotonic or classify patients according to volume status. Second, we did not know with certainty the cause of hyponatremia or whether the hyponatremia was acute, subacute or chronic. Hyponatremia is an etiologically heterogeneous condition, with hypo-, eu- and hypervolemic as well as drug-induced and disease-related phenotypes that differ in both prognosis and management. With this pragmatic analysis in multimorbid older adults with polypharmacy, we still captured a usually underrepresented population in clinical research. While we were unable to directly classify phenotypes, we attempted to mitigate this limitation by adjusting for relevant comorbidities and by performing sensitivity analyses excluding patients on drugs known to cause hyponatremia and additionally adjusting for infection, heart failure and malignancy. These analyses did not materially change the results. Third, blood glucose values were not available in all participants, which precluded a glucose correction of sodium values. This might have led to some cases of pseudohyponatremia in our study population. In a random subsample of 100 participants with documented blood glucose values, corrections were infrequent and small, clustering around the 135 mmol/L threshold, but the limited data preclude firm conclusions. Fourth, while we performed a sensitivity analysis in which we excluded patients taking drugs known to cause hyponatremia, as mentioned above, there are drugs where the association with sodium is unclear or unknown. We cannot exclude the possibility that other drugs may have had a significant influence on sodium values. Fifth, while we adjusted for comorbidities using the weighted Charlson comorbidity index, we acknowledge that not all chronic conditions contribute equally to clinical severity in older adults. Grouping comorbidities by severity could offer added nuance, but would require arbitrary stratification not pre-specified in our methodology. We partly addressed this limitation by performing sensitivity analyses adjusting for infection, heart failure and malignancy, as mentioned above. These were chosen based on their potential to influence both sodium levels and clinical outcomes. Infection may lead to transient hyponatremia with good prognosis if treated successfully—particularly relevant given our population included only survivors of the index hospitalization—while malignancy and heart failure are chronic conditions that could independently contribute to both hyponatremia and increased mortality [30,31]. Regarding malignancies, information on the specific types of malignancies was not available in our dataset, which precluded more detailed analyses; even if available, subgroup numbers would likely have been too small to allow meaningful stratification. Nonetheless, there is the need for future studies to incorporate more refined measures of multimorbidity severity. Sixth, we did not have sodium values at the time of death or readmission. Finally, as we had predominantly Caucasian participants, the results may not be generalizable to non-Caucasian populations.

Our findings show that multimorbid older adults with hyponatremia at admission have a markedly higher risk of 1-year mortality, highlighting serum sodium as a potentially valuable prognostic marker in this population. For clinicians, this reinforces the importance of recognizing hyponatremia as more than an incidental laboratory finding—it may serve as a signal for closer monitoring or further evaluation of underlying disease severity. Given the observational design of our study, causality cannot be established. Sodium most likely functions as a marker of underlying illness, but it is also possible that hyponatremia itself contributes negatively to survival, as suggested by previous work [36,45]. However, as multimorbidity encompasses a wide range of conditions with varying clinical implications, future research should aim to identify which patient profiles most strongly contribute to the observed mortality risk. The potentially independent association between hyponatremia and mortality should be further explored in a prospective manner with assessment of the most likely causes of hyponatremia and whether the condition is acute or chronic and with repeated sodium measurements during follow-up. In addition, sufficiently powered studies are needed to determine whether treatment of hyponatremia can significantly reduce mortality.

## 5. Conclusions

Among multimorbid older patients, hyponatremia at admission was associated with increased 1-year mortality, with increasing risk for lower sodium values, but not with 1-year readmissions. These findings underscore the potential value of sodium measurement in identifying at-risk older patients upon hospital admission. While sodium likely reflects underlying illness, it may also exert independent adverse effects on survival, which should be addressed in future prospective and interventional studies. Recognizing hyponatremia as a—potentially independent—risk factor could support more informed clinical decisions and targeted monitoring strategies in multimorbid older populations.

## Figures and Tables

**Table 1:** Baseline characteristics of OPERAM participants with admission serum sodium.

Characteristics	Hyponatremia (n = 401)	Normonatremia (n = 1534)
Serum sodium (mmol/L)		
median (IQR)	132 (129–133)	139 (137–141)
range	104–134	135–145
Age (years)		
median (IQR)	80 (75–85)	78 (74–84)
range	70–95	70–99
Female sex—n (%)	205 (51.1)	661 (43.1)
Education—n (%) *		
less than high school	132 (33.4)	446 (29.4)
high school	178 (45.1)	703 (46.3)
university	85 (21.5)	368 (24.3)
Nursing home—n (%) **	29 (7.2)	156 (10.2)
Barthel index—median (IQR) ^†^	90 (75–100)	95 (80–100)
History of falls in the last year—n (%)	166 (41.4)	577 (37.6)
Comorbidities—median (IQR)	11 (8–16)	11 (8–16)
acute myocardial infarction—n (%)	35 (8.7)	168 (11)
congestive heart failure—n (%)	108 (26.9)	411 (26.8)
peripheral vascular disease—n (%)	69 (17.2)	317 (20.7)
cerebrovascular disease—n (%)	126 (31.4)	424 (27.6)
dementia—n (%)	19 (4.7)	98 (6.4)
COPD—n (%)	99 (24.7)	416 (27.1)
rheumatic disease—n (%)	30 (7.5)	109 (7.1)
peptic ulcer disease—n (%)	14 (3.5)	68 (4.4)
liver disease—n (%)	21 (5.2)	82 (5.3)
hemiplegia or paraplegia—n (%)	12 (3)	46 (3)
renal disease—n (%)	99 (24.7)	455 (29.7)
any malignancy—n (%)	111 (27.7)	407 (26.5)
metastatic solid tumor—n (%)	17 (4.2)	53 (3.5)
AIDS/HIV—n (%)	0 (0)	0 (0)
Current smoking	27 (6.7)	128 (8.3)
Alcohol consumption (units/week)— median (p25–p75)	0 (0–4)	0 (0–4)
No. of concomitant medications—median (IQR)	9 (7–13)	10 (7–13)
thiazides—n (%)	68 (17)	225 (14.7)
SSRIs—n (%)	54 (13.5)	232 (15.1)
other antidepressants ^}^—n (%)	73 (18.2)	258 (16.8)
bisphosphonates ^}^—n (%)	31 (7.7)	105 (6.8)
vasopressin ^}^—n (%)	1 (0.2)	2 (0.1)
antipsychotic drugs ^}^—n (%)	14 (3.5)	51 (3.3)
antiepileptic drugs ^}^—n (%)	18 (4.5)	47 (3.1)
antidiabetic drugs ^}^—n (%)	0 (0)	4 (0.3)
anticancer drugs ^}^—n (%)	10 (2.5)	49 (3.2)

Abbreviations: IQR: interquartile range; COPD: chronic obstructive pulmonary disease; No.: number; SSRIs: selective serotonin reuptake inhibitors. * n = 23 missing (6 in hyponatremia and 17 in normonatremia). ** Any time spent in a nursing home in the last six months before randomization. ^†^ The Barthel index is on a scale from 0 to 100, with higher scores indicating more functional independence in the domains of personal care and mobility. ^}^ Other antidepressants known to cause hyponatremia: trazodone, mirtazapine, venlafaxine, duloxetine, amitriptyline, doxepin; vasopressin and vasopressin analogues; antipsychotics: phenothiazines, butyrophenone derivatives; antiepileptic drugs: carboxamide derivatives, valproic acid, lamotrigine; antidiabetic drugs: tolbutamide; anticancer drugs: cyclophosphamide, ifosfamide, methotrexate.

**Table 2:** Mortality and readmission in older multimorbid adults with hyponatremia vs. normonatremia at admission across sodium categories.

	n ^†^	No. of Events (%) ^†^	Hazard Ratio *	95% CI	*p*
1-year mortality	**1935**	**364 (18.8)**			
Normal sodium°	1534	266 (17.3)	Ref.		
Hyponatremia	401	98 (24.4)	**1.41**	**1.11–1.78**	**0.005**
-mild °	283	66 (23.3)	1.31	1.00–1.73	
-moderate °	82	19 (23.1)	1.33	0.83–2.13	0.001 ^П^
-severe °	36	13 (36.1)	2.64	1.50–4.67	
30-day mortality	**1935**	**89 (4.6)**			
Normal sodium	1534	66 (4.3)	Ref.		
Hyponatremia	401	23 (5.7)	**1.20**	**0.74–1.94**	**0.46**
-mild °	283	14 (4.9)	1.04	0.58–1.86	
-moderate °	82	4 (4.9)	0.97	0.35–2.68	0.02 ^П^
-severe °	36	5 (13.9)	3.36	1.31–8.62	
1-year readmission	**1935**	**943 (48.7)**			
Normal sodium	1534	756 (49.3)	Ref.		
Hyponatremia	401	187 (46.6)	**0.94**	**0.79–1.11**	**0.46**
-mild °	283	135 (47.7)	0.94	0.77–1.15	
-moderate °	82	35 (42.7)	0.87	0.60–1.27	0.98 ^П^
-severe °	36	17 (47.2)	1.03	0.64–1.69	
30-day readmission	**1935**	**210 (10.9)**			
Normal sodium	1534	163 (10.6)	Ref.		
Hyponatremia	401	47 (11.7)	**1.11**	**0.78–1.59**	**0.55**
-mild °	283	31 (11.0)	1.04	0.69–1.56	
-moderate °	82	11 (13.4)	1.27	0.70–2.31	0.40 ^П^
-severe °	36	5 (13.9)	1.36	0.56–3.28	

Abbreviations: No.: number, Ref.: reference, CI: confidence interval. * Hazard ratio comparing participants with hyponatremia (<135 mmol/L) with participants with normal serum sodium levels. In bold the overall number of participants, events and the main analyses comparing the outcome in participants with normal sodium versus hyponatremia overall. Higher numbers indicate higher likelihood of an outcome for participants with hyponatremia. The results for mortality were obtained using a mixed-effects survival model with adjustment for age, sex, intervention, weighted Charlson comorbidity index and site. Cluster was added as a random effect. The results for readmission were obtained using a competing risk model with death as the competing risk, with otherwise the same adjustment variables. ^†^ n/No. of events: the number of participants overall and in hyponatremia categories, and the number of events (death or at least one readmission) in the corresponding participants. ° Sodium categories used were mild (1340–130 mmol/L), moderate (129–125 mmol/L) and severe (<125 mmol/L) hyponatremia in comparison to normal sodium values (135–145 mmol/L). ^П^ Linear trend across sodium categories.

**Table 3:** Mortality and readmission in multimorbid older adults with sodium trajectories: hyponatremia at admission and/or discharge vs. normal sodium values at admission and discharge measurement.

	n ^†^	No. of Events (%) ^†^	Hazard Ratio *	95% CI	*p*
1-year mortality	**1782**	**303 (17.0)**			
Normal sodium	1347	205 (15.2)	Ref.		
Hyponatremia	435	98 (22.5)	**1.47**	**1.15–1.89**	**0.002**
-resolved °	227	44 (19.4)	1.18	0.85–1.64	0.33
-persistent °	151	41 (27.2)	2.03	1.44–2.86	<0.001
-acquired °	57	13 (22.8)	1.43	0.81–2.53	0.21
30-day mortality	**1782**	**43 (2.4)**			
Normal sodium	1347	31 (2.3)	Ref.		
Hyponatremia	435	12 (2.8)	**1.06**	**0.54–2.07**	**0.87**
-resolved °	227	5 (2.2)	0.85	0.33–2.81	0.73
-persistent °	151	5 (3.3)	1.26	0.48–3.30	0.63
-acquired °	57	2 (3.5)	1.39	0.33–5.85	0.66
1-year readmission	**1782**	**886 (49.7)**			
Normal sodium	1347	675 (50.1)	Ref.		
Hyponatremia	435	211 (48.5)	**0.81**	**0.67–0.99**	**0.04**
-resolved °	227	105 (46.3)	0.82	0.64–1.04	0.10
-persistent °	151	78 (51.7)	0.73	0.52–1.04	0.08
-acquired °	57	28 (49.1)	1.02	0.64–1.60	0.95
30-day readmission	**1782**	**181 (10.2)**			
Normal sodium	1347	130 (9.7)	Ref.		
Hyponatremia	435	51 (11.7)	**1.25**	**0.87–1.79**	**0.23**
-resolved °	227	27 (11.9)	1.25	0.76–2.06	0.38
-persistent °	151	17 (11.3)	1.21	0.71–2.05	0.49
-acquired °	57	7 (12.3)	1.34	0.66–2.75	0.42

° Sodium trajectories used were resolved hyponatremia (sodium < 135 mmol/L at admission, normal sodium 135–145 mmol/L at discharge), persistent hyponatremia (sodium < 135 mmol/L at admission and discharge) and acquired hyponatremia (normal sodium at admission, <135 mmol/L at discharge), compared to two normal sodium values. Hyponatremia was sodium <135 mmol/L at either time point. * Hazard ratio comparing participants with hyponatremia (<135 mmol/L) with participants with two normal serum sodium values. In bold the overall number of participants, events and the main analyses comparing the outcome in participants with normal sodium at both admission and discharge versus hyponatremia at at least one timepoint. Higher numbers indicate higher likelihood of an outcome for participants with hyponatremia. The results for mortality were obtained using a mixed-effects survival model with adjustment for age, sex, intervention, weighted Charlson comorbidity index and site. Cluster was added as a random effect. The results for readmission were obtained using a competing risk model with death as the competing risk, with otherwise the same adjustment variables. ^†^ n/No. of events: number of participants overall with sodium measurement at admission and discharge, participants with normal sodium at admission and discharge or hyponatremia (<135 mmol/L) at admission and/or discharge, and the number of events (death or at least one readmission) in the corresponding participants. For trajectories see °.

## Data Availability

Data will be made available for scientific purposes for researchers whose proposed use of the data has been approved by a publication committee. Data and documentation will be made available through a secure file exchange platform after approval of proposal and after a data transfer agreement is signed (which defines obligations that the data requester must adhere to with regard to privacy and data handling). For data access, please contact operam@biham.unibe.ch.

## Supplementary Materials

The following supporting information can be downloaded at https://www.mdpi.com/article/10.3390/jcm14207146/s1: Table S1: ICD-codes for infection; Table S2: List of drugs known to cause hyponatremia, excluded in sensitivity analysis; Table S3: Mortality and readmission in older multimorbid adults with hyponatremia vs. normonatremia at admission, with adjustment for the presence of heart failure, malignancy and infection; Table S4: Mortality and readmission in older multimorbid adults with hyponatremia vs. normonatremia at discharge; Figure S1: Sodium and 1-year mortality via restricted cubic spline.

## Abbreviations

The following abbreviations are used in this manuscript:

OPERAM	OPtimising thERapy to prevent Avoidable hospital admissions in Multimorbid older adults
HR	Hazard ratio
CI	Confidence interval
SHR	Subhazard ratio
e.g.	Example given
