# Supplementary material for: Association Between Hyponatremia and Mortality and Readmission in Multimorbid Older Adults—A Cohort Study
Source: J Clin Med. 2025 Oct 10;14(20):7146. doi: 10.3390/jcm14207146 (PMC12565651; doi:10.3390/jcm14207146)
Supplement: Supplementary file 1 [file jcm-14-07146-s001.zip › jcm-3803268-supplementary.pdf]

Multidisciplinary
Digital Publishing
Institute

MDPI AG
Kandererstrasse 25
CH-4057 Basel
Switzerland

Tel. +41 61 683 77 34
Fax +41 61 302 89 18
www.mdpi.com

JCM — Change of Authorship Form

(Must be Completed and Signed by ALL Authors)

Manuscript: ID: **jcm-3803268**

Manuscript: Title: Association between hyponatremia and mortality and readmission in multimorbid older adults – a cohort study

Original Authorship

LIST ALL AUTHORS in the same order as the original (first) submission. For more than 10 use an extra sheet.

	name	Affiliation
author (1)	Seraina Netzer	Department of General Internal Medicine, Inselspital, Bern University Hospital, University of Bern, Bern, Switzerland<br>Institute of Primary Health Care (BIHAM), University of Bern, Bern, Switzerland
author (2)	Stéphanie Baggio 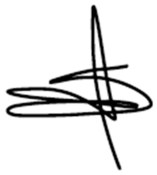	Institute of Primary Health Care (BIHAM), University of Bern, Bern, Switzerland<br>Laboratory of Population Health, University of Fribourg, Fribourg, Switzerland<br>New: Institute of Psychology, University of Lausanne, Lausanne, Switzerland
author (3)	Viktoria Gastens	Institute of Primary Health Care (BIHAM), University of Bern, Bern, Switzerland<br>Laboratory of Population Health, University of Fribourg, Fribourg, Switzerland
author (4)	Benoît Boland	Department of Geriatric Medicine, UC Louvain, St-Luc university hospital, Brussels, Belgium<br>Institute of Health and Society (IRSS), Université Catholique de Louvain, Brussels, Belgium
author (5)	Carole Elodie Aubert	Department of General Internal Medicine, Inselspital, Bern University Hospital, University of Bern, Bern, Switzerland<br>Institute of Primary Health Care (BIHAM), University of Bern, Bern, Switzerland
author (6)	Corlina J.A. Huibers	Department of Geriatric Medicine, University Medical Centre Utrecht, Utrecht University, Utrecht, The Netherlands
author (7)	Wilma Knol	Department of Geriatric Medicine, University Medical Centre Utrecht, Utrecht University, Utrecht, The Netherlands

author (8)	Anne Spinewine	Department of Pharmacy, CHU UCL Namur, Yvoir, Belgium<br>Louvain Drug Research Institute, Université catholique de Louvain, Brussels, Belgium
author (9)	Denis O'Mahony	Department of Medicine, School of Medicine, University College Cork, Ireland
author (10)	Drahomir Antonin Aujesky	Department of General Internal Medicine, Inselspital, Bern University Hospital, University of Bern, Bern, Switzerland
author (11)	Mirjam Christ-Crain	Division of Endocrinology, Diabetes and Metabolism, University Hospital Basel; Department of Clinical Research, University of Basel, Basel, Switzerland
author (12)	Douglas C. Bauer	Department of Medicine and Department of Epidemiology and Biostatistics, University of California, San Francisco, San Francisco, CA, USA
author (13)	Nicolas Rodondi	Department of General Internal Medicine, Inselspital, Bern University Hospital, University of Bern, Bern, Switzerland<br>Institute of Primary Health Care (BIHAM), University of Bern, Bern, Switzerland
author (14)	Martin Feller	Institute of Primary Health Care (BIHAM), University of Bern, Bern, Switzerland

New Authorship

All authors must sign below agreeing to the new changes in authorship. The authorship order must match the new title page of the manuscript. Signatures below certify compliance with the author responsibilities on the next page. List ALL AUTHORS in the same order as the new version.

	name	affiliation	Signature&Date
author (1)	Seraina Netzer	Department of General Internal Medicine, Inselspital, Bern University Hospital, University of Bern, Bern, Switzerland<br>Institute of Primary Health Care (BIHAM), University of Bern, Bern, Switzerland	24.09.2025
author (2)	Viktoria Gastens	Institute of Primary Health Care (BIHAM), University of Bern, Bern, Switzerland<br>Laboratory of Population Health, University of Fribourg, Fribourg, Switzerland	
author (3)	Benoît Boland	Department of Geriatric Medicine, UC Louvain, St-Luc university hospital, Brussels, Belgium<br>Institute of Health and Society (IRSS), Université Catholique de Louvain, Brussels, Belgium	

author (4)	Carole Elodie Aubert	Department of General Internal Medicine, Inselspital, Bern University Hospital, University of Bern, Bern, Switzerland<br><br>Institute of Primary Health Care (BIHAM), University of Bern, Bern, Switzerland	
author (5)	Corlina J.A. Huibers	Department of Geriatric Medicine, University Medical Centre Utrecht, Utrecht University, Utrecht, The Netherlands	
author (6)	Wilma Knol	Department of Geriatric Medicine, University Medical Centre Utrecht, Utrecht University, Utrecht, The Netherlands	24-9-2025
author (7)	Anne Spinewine	Department of Pharmacy, CHU UCL Namur, Yvoir, Belgium<br><br>Louvain Drug Research Institute, Université catholique de Louvain, Brussels, Belgium	24/09/2025
author (8)	Denis O'Mahony	Department of Medicine, School of Medicine, University College Cork, Ireland	
author (9)	Drahomir Antonin Aujesky	Department of General Internal Medicine, Inselspital, Bern University Hospital, University of Bern, Bern, Switzerland	23.09.25
author (10)	Mirjam Christ-Crain	Division of Endocrinology, Diabetes and Metabolism, University Hospital Basel; Department of Clinical Research, University of Basel, Basel, Switzerland	30.09.2025
author (11)	Douglas C. Bauer	Department of Medicine and Department of Epidemiology and Biostatistics, University of California, San Francisco, San Francisco, CA, USA	September 22, 2025
author (12)	Nicolas Rodondi	Department of General Internal Medicine, Inselspital, Bern University Hospital, University of Bern, Bern, Switzerland<br><br>Institute of Primary Health Care (BIHAM), University of Bern, Bern, Switzerland	25.09.2025
author (13)	Martin Feller	Institute of Primary Health Care (BIHAM), University of Bern, Bern, Switzerland	

Please list all the author's Contribution here:

Author's Contribution
author (1) Conceptualisation; Data curation; Formal analysis; Funding acquisition; Methodology; Writing – original draft
author (2) Data curation; Writing – review & editing
author (3) Conceptualisation; Resources; Writing – review & editing

author (4) Writing – review & editing
author (5) Resources; Writing – review & editing
author (6) Writing – review & editing
author (7) Resources; Writing – review & editing
author (8) Resources; Writing – review & editing
author (9) Conceptualisation; Funding acquisition; Writing – review & editing
author (10) Conceptualisation; Funding acquisition; Methodology; Writing – review & editing
author (11) Conceptualisation; Funding acquisition; Methodology; Writing – review & editing
author (12) Conceptualisation; Funding acquisition; Investigation; Methodology; Resources; Supervision; Writing – review & editing
author (13) Conceptualisation; Funding acquisition; Investigation; Methodology; Supervision; Writing – review & editing
